# Applying multi‐omics toward tumor microbiome research

**DOI:** 10.1002/imt2.73

**Published:** 2023-01-09

**Authors:** Nan Zhang, Shruthi Kandalai, Xiaozhuang Zhou, Farzana Hossain, Qingfei Zheng

**Affiliations:** ^1^ Department of Radiation Oncology, College of Medicine The Ohio State University Columbus Ohio USA; ^2^ Center for Cancer Metabolism, Ohio State University Comprehensive Cancer Center ‐ James Cancer Hospital and Solove Research Institute The Ohio State University Ohio Columbus USA; ^3^ Department of Biological Chemistry and Pharmacology, College of Medicine The Ohio State University Columbus Ohio USA

**Keywords:** cancer biology, host‐microbe interaction, multi‐omics, tumor microbiome, tumor microenvironment

## Abstract

Rather than a “short‐term tenant,” the tumor microbiome has been shown to play a vital role as a “permanent resident,” affecting carcinogenesis, cancer development, metastasis, and cancer therapies. As the tumor microbiome has great potential to become a target for the early diagnosis and treatment of cancer, recent research on the relevance of the tumor microbiota has attracted a wide range of attention from various scientific fields, resulting in remarkable progress that benefits from the development of interdisciplinary technologies. However, there are still a great variety of challenges in this emerging area, such as the low biomass of intratumoral bacteria and unculturable character of some microbial species. Due to the complexity of tumor microbiome research (e.g., the heterogeneity of tumor microenvironment), new methods with high spatial and temporal resolution are urgently needed. Among these developing methods, multi‐omics technologies (combinations of genomics, transcriptomics, proteomics, and metabolomics) are powerful approaches that can facilitate the understanding of the tumor microbiome on different levels of the central dogma. Therefore, multi‐omics (especially single‐cell omics) will make enormous impacts on the future studies of the interplay between microbes and tumor microenvironment. In this review, we have systematically summarized the advances in multi‐omics and their existing and potential applications in tumor microbiome research, thus providing an omics toolbox for investigators to reference in the future.

## Highlights

- The tumor microbiome plays a significant role in carcinogenesis, cancer development, metastasis, and cancer therapies.
- The tumor microbiome has great potential to become a target for early diagnosis and treatment of cancer.
- Genomics, transcriptomics, proteomics, and metabolomics are powerful approaches for tumor microbiome studies, by facilitating an understanding of different levels of the central dogma.
- Multi‐omics will make enormous impacts on future research of the tumor microbiome.
- The development of single‐cell multi‐omics technologies will motivate the progression of the tumor microbiome field.

## Graphical abstract

In this review, we have systematically summarized the advances of multi‐omics and their existing and potential applications in tumor microbiome research, thus providing an omics toolbox for investigators to reference in the future.

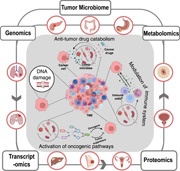

## INTRODUCTION

**Figure 1:**
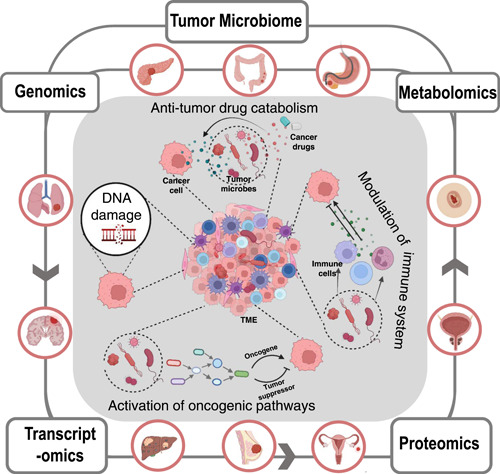
An integrated model showing of the roles of microorganisms in carcinogenesis and tumor development, various types of tumors that the tumor microbiome has been linked to, and multi‐omics research methods of the tumor microbiome. TME, tumor microenvironment.

The earliest known historical records linking cancer and microbes can be traced back to over a century ago [1]. There are currently about 10^12^ known microbial species on Earth [1, 2], with only 11 having been declared to be “oncomicrobes” (or pathogens known to be carcinogenic to humans) by the International Agency for Research on Cancer (IARC). These tumor microbes cause about 2.2 million cases every year or ~13% of cancer cases worldwide. Furthermore, researchers have found that live microbes exist in many kinds of tumors (Figure 1), including breast cancer [3], colorectal cancer [4, 5], hepatocellular carcinoma [6, 7], pancreatic cancer [8, 9], skin cancer [10], renal cell carcinoma [11], gastric cancer [12], lung cancer [13, 14], prostate cancer [15–17], nasopharyngeal carcinoma [18], and so forth. Notably, microbes in the same type of tumors have striking similarities [19]. Intratumoral microbes have been found to affect the development and treatment of tumors through diverse mechanisms, such as DNA damage, oncogenic pathway activation, antitumor drug catabolism, and immune system modulation (Figure 1) [19–25].

Research advances have begun to uncover a sliver of the full scope in understanding how the tumor microbiome influences cancer [26]. One well‐established mechanism of these microorganisms is the release of genotoxins that are capable of inducing oncogenic DNA mutations, including colicin (DNA alkylator) from *Escherichia coli* [27], cytolethal distending toxins (deoxyribonuclease activator) from Gram‐negative bacteria (e.g., *Campylobacter jejuni*) [28], and toxins that act as reactive oxygen species (ROS) producers from enterotoxigenic *Bacteroides fragilis* [29]. Furthermore, many regulatory pathways related to cancer pathogenesis can be affected by microbes. For instance, *Helicobacter pylori*, one of the aforementioned 11 declared oncomicrobes, has been shown to cause carcinogenesis, by inducing inflammation and affecting key intracellular signal pathways that regulate the growth and proliferation of mucosal cells [30]. In addition to influencing tumorigenesis, the microbial community has been implicated in cancer treatment approaches, through the metabolism of anti‐tumor drugs. Gemcitabine, a chemotherapeutic drug commonly used to treat pancreatic ductal adenocarcinoma, can be metabolized to an inactive state by intratumoral bacteria, thereby resulting in cancer drug resistance [31]. Moreover, many microorganisms can affect cancer development and therapies by affecting host immunosurveillance [32]. Therefore, the tumor microbiome is gradually becoming a new focus in the early diagnosis and treatment (especially immuno‐oncology) of cancer [33, 34].

Even though research into the tumor microbiome has made great progress, a number of challenges in this emerging area still remain, such as the low biomass of intratumoral bacteria, unculturable character of some microbial species, and heterogeneity of the tumor microenvironment (TME) [35–37]. Thus, our understanding of the tumor microbiome is still far from sufficient, mainly due to the limits in research technologies. As the tumor microbiome is becoming more and more significant in both basic and translational research, the development of new methods with high spatial and temporal resolution is required. Recently, the integrated application of multi‐omics technologies (combining genomics, transcriptomics, proteomics, and metabolomics) has shown great power in biomedical research and facilitated the understanding of many previously elusive biological processes [38]. To reveal the pathophysiological functions of the tumor microbiome in cancer development and therapies on different levels of the central dogma (Figure 1), multi‐omics (especially single‐cell omics) approaches have begun to be developed and applied [39, 40]. Therefore, in this review, we will systematically summarize the multi‐omics techniques that can be utilized for tumor microbiome research and present our views on the current advantages and limitations of these methodologies.

## GENOMICS AND TRANSCRIPTOMICS FOR TUMOR MICROBIOME STUDIES

**Figure 2:**
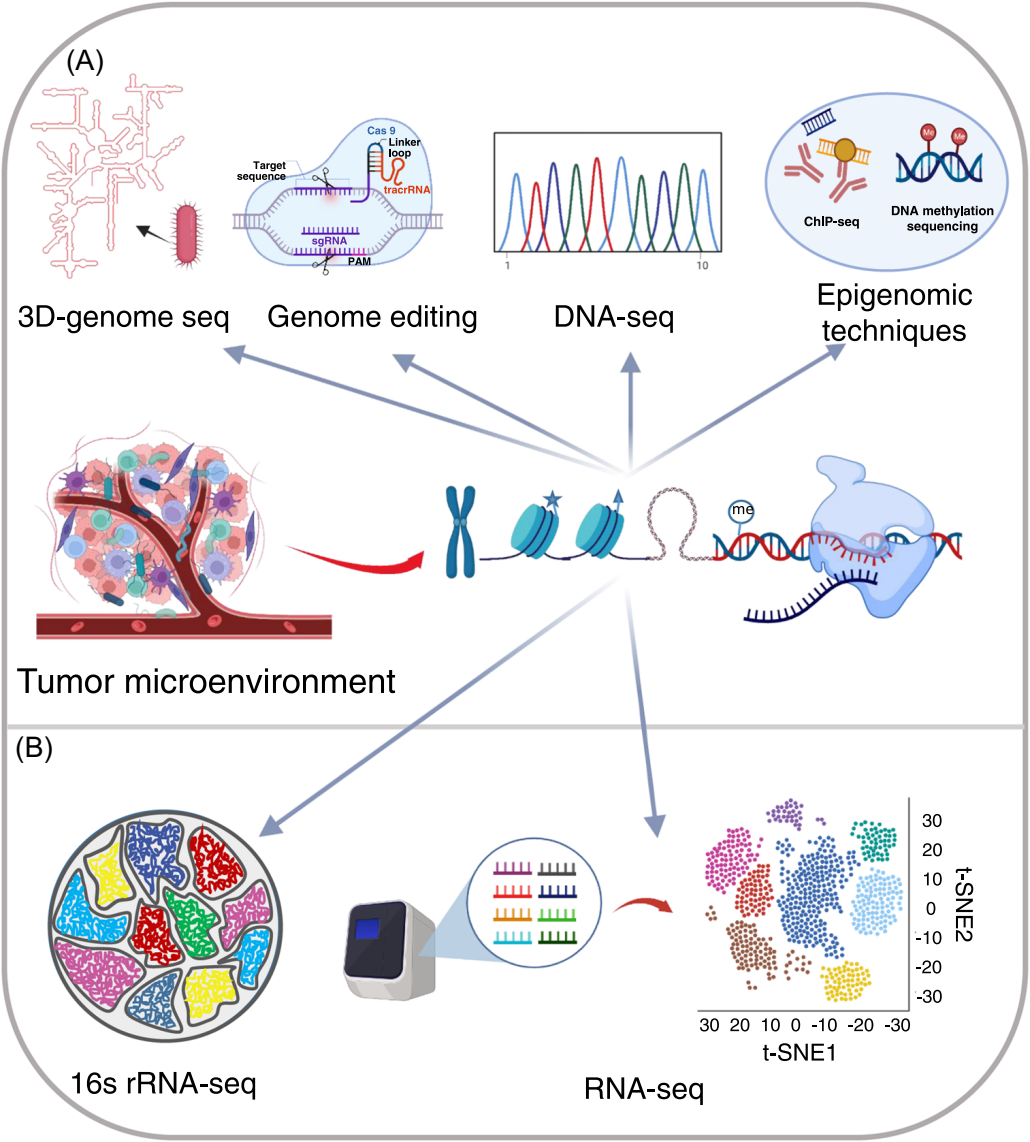
Techniques for genome and transcriptome research of the tumor microenvironment. (A) Techniques for metagenomic sequencing. CHIP‐seq, chromatin immunoprecipitation sequencing; DNA‐seq, DNA sequencing. 16S rRNA‐seq, 16S ribosomal RNA sequencing; RNA‐seq, RNA sequencing.

Genomics and transcriptomics focus on nucleic acids (i.e., DNA and RNA), especially those involved in the regulation of gene expression. At present, a large number of research techniques have been developed to study the genomes and transcriptomes of diverse biological systems. These techniques of note include high throughput sequencing and gene editing (Figure 2). Currently, the most widely employed sequencing techniques include three next‐generation sequencing (NGS) systems (i.e., Illumina, Ion Torrent, BGI) and two third‐generation sequencing techniques (i.e., PacBio and Nanopore) [41]. These sequencing approaches are the basis for the development of a variety of genomic and transcriptomic research methodologies, including DNA sequencing [15], RNA sequencing [42], 16S rRNA sequencing [43], epigenetic techniques (e.g., ChIP‐seq and DNA/RNA methylation sequencing) [44–46], and three‐dimensional genomic technologies [47]. Moreover, recent research advances of gene editing tools have enabled multi‐gene manipulation on a genome‐wide level [48]. As these technologies can easily target nucleic acids, they have become important tools for research into the tumor microbiome and TME, greatly accelerating the identification and traceability of tumor microbes, and helping to reveal how tumor microbes, cancer cells, and the immune system interact [7, 33, 49, 50].

### DNA sequencing (DNA‐seq)

DNA‐seq is one of the most important approaches in microbial genome research, which can be applied to analyze all of the genes of complex samples, such as the human microbiome. DNA‐seq is often used for three types of genomic analyses: whole‐genome sequencing (WGS), whole‐exome sequencing (WES), and targeted sequencing [51, 52]. With the rapid development of DNA technologies, DNA‐seq is becoming a powerful tool to study unculturable microbes, including those in the human microbiome [53, 54]. The sequencing and heterologous expression of genomic libraries significantly facilitates our understanding of metabolic pathways and the physiology of the tumor microbiome [55, 56].

### 16S rRNA sequencing

16S rRNA sequencing is a sequencing technology that is specifically focused on the 16 S component of the 30 S ribosomal subunit expressed by bacteria and archaea. This sequence contains alternating highly conserved and hypervariable regions [57]. The conserved region can be used to design universal primers for the amplification of target fragments, while analyses of the hypervariable region can be employed to identify specific bacterial species, though some annotations may be below the species level [58]. In the tumor microbiome field, 16 S rRNA sequencing is mainly applied to study the species composition in communities, evolutionary relationships between species, and diversity of microbial communities [19].

### RNA sequencing (RNA‐seq)

RNA‐seq is a transcriptome sequencing method, where high‐throughput sequencing methodologies are utilized to study the populations of diverse RNA in complex samples, including messenger RNA (mRNA), transfer RNA (tRNA), noncoding RNA (ncRNA), microRNA (miRNA), etc. Over the past decades, RNA‐seq technologies have developed rapidly and become an indispensable tool to analyze gene expression on the transcriptome level [59]. The application ranges of RNA‐seq have grown with the development of NGS. In the field of RNA biology, RNA‐seq has been widely applied to study single‐cell gene transcription, protein expression, and RNA structures [60, 61]. The advances of RNA‐seq technologies have also facilitated the emergence and development of spatial transcriptomics [62]. Direct long‐read or full‐length RNA‐seq methods, as well as better data analysis methods, have enabled researchers to better understand RNA biology, including the machinery of the transcription process and the impacts of folding and intermolecular interactions on RNA function [63–65]. Overall, RNA‐seq is a powerful tool to investigate the mutual influences of cancer cells and tumor microbiome on each other's pathways by analyzing at the level of gene transcription.

### Epigenomic techniques

Epigenetics has been referred to as the investigation of phenotypic changes that do not involve alterations in the DNA sequence. Distinct from genetic regulation, typical epigenetic changes (including DNA/RNA modifications and histone posttranslational modifications) are reversible and do not influence cellular genomic sequences. In eukaryotic cells, nucleosomes are the basic repeating unit of chromatin organization and are comprised of 147 base pairs of DNA spooled 1.5 times around an octamer of core histones (i.e., two copies of H2A, H2B, H3, and H4). The dynamics of covalent modifications on DNA, RNA, and histones are a key mechanism of epigenetic regulation that controls the activation and suppression of specific genes. Epigenetic regulation plays an essential role in regulation of chromatin structure and function during replication, transcription, and repair of DNA damage, which have close relevance to many human diseases, especially cancer [66].

Specifically, DNA methylation alters the genetic transcription without changing the genome sequence, which may result in tumorigenesis when tumor suppressor genes are turned off [67]. A large number of studies have shown that aberrant methylation of DNA is closely related to the occurrence and development of diverse types of tumors [68, 69]. Therefore, changes in DNA methylation levels have been detected as biomarkers for cancer diagnosis [70]. Currently, multiple DNA methylation sequencing techniques have been developed, including whole genome bisulfite sequencing (WGBS) [71], reduced representation bisulfite sequencing (RRBS) [72], oxidative bisulfite sequencing (oxBS‐seq) [73], and cell‐free methylated DNA immunoprecipitation and high‐throughput sequencing (cfMeDIP‐seq) [74].

On the other hand, histone posttranslational modifications (PTMs) include methylation, phosphorylation, acetylation, crotonylation, ubiquitination, glycosylation, glycation, and ADP ribosylation [75]. Various histone PTMs are believed to be involved in the emergence and development of cancers [76]. For example, methylglyoxal synthases (MGSs) derived from microorganisms can biosynthesize excess amount of methylglyoxal in TME [77, 78]. Histone MGO‐glycation has been shown to influence tumor development, by affecting the three‐dimensional architecture of chromatin [79, 80].

Chromatin immunoprecipitation sequencing (ChIP‐seq) is one of the most commonly used methods used to study the effects of histone modifications on gene transcription [81, 82], though it was initially developed to study the interactions between proteins and DNA [83]. Chromatin immunoprecipitation (ChIP), also known as binding site analysis, is a powerful tool to study these interactions in vivo and has been crucial to better understanding epigenetic changes to the genome [84]. ChIP‐seq, which combines ChIP with second‐generation DNA sequencing, can efficiently detect the DNA binding sites of specific histone modifications and transcription factors [85]. The principle of ChIP‐seq is as follows: DNA fragments bound with a protein of interest are enriched and immunoprecipitated by ChIP before being purified, and thereafter, a library is constructed. The enriched DNA fragments are then subjected to high‐throughput sequencing. By applying this method, researchers have accurately located sequences in the genome that interact with specific histones [86] and transcription factors [87]. Importantly for tumor microbiome research, ChIP‐seq is suitable for the (epi)genomic studies of both host and microbe cells.

### Three‐dimensional (3D) genomics

The 3D genomics, also known as spatial genomics, is based on the basic information of the 1D genome sequence and studies the 3D structure of genome, as well as the mechanisms of transcriptional regulation mediated by different elements (including transcription factors and DNA/RNA‐binding proteins). In recent years, the technologies developed in this field mainly include Hi‐C (high‐throughput/resolution chromosome conformation capture) [88, 89], Micro‐C (micrococcal nuclease‐based analysis of chromosome folding) [90], Micro‐C XL (micrococcal nuclease‐based analysis of chromosome folding using long x‐linkers) [91], ChIA‐PET (chromatin interaction analysis by paired‐end tag sequencing) [92], and HiChIP (in situ Hi‐C followed by chromatin immunoprecipitation) [93]. As related to tumor microbiome research, while most of these approaches above are focused on eukaryotic genomes of host cells, Hi‐C technology has emerged recently as a powerful tool to investigate bacterial chromosomes, enabling the physical proximity of DNA sequences in a sample to be assayed [94, 95].

### Genome editing

Gene editing (also known as genetic modification or genetic engineering) mainly refers to the use of biochemical approaches to modify genomic sequences. This is often done by introducing the target gene fragment to the genome or deleting the specific gene region from the genome, so as to change the genotype of the host cell or strengthen the original genotype [96]. As there is a long history of gene editing, many elegant tools have been developed as “genetic scalpels,” including artificial restriction enzymes [97], zinc finger nucleases (ZFNs) [98], transcription activator‐like effector nucleases (TALENs) [99], clustered regularly interspaced short palindromic repeats (CRISPR) and CRISPR‐associated protein 9 (Cas9) [100–102], CRISPR base editors [103], prime editing systems [104], and programmable addition through site‐specific targeting elements (PASTE) [105]. At present, the CRISPR‐Cas9 technology and the systems derived from it are the mainstream technologies most widely utilized in genome editing [106]. Furthermore, Cas9 endonuclease dead (also known as dead Cas9 or dCas9) is a mutant form of Cas9, that can no longer act as an endonuclease, due to point mutations in its endonuclease domains [107]. dCas9, which serves as a gene location guide, is now widely used in the genome editing of different biological systems through the fusion expression with other functional enzymes (such as cytidine deaminases [108], adenine deaminases [109], and epigenetic writer/eraser enzymes [110, 111]) and other resulting emerging technologies (e.g., epigenome editing) [112, 113].

As for tumor microbiome research, genome editing has been widely applied toward studying both host cells [114] and microbes [115]. For example, it has been reported that the genomic composition of intestinal microorganisms varies widely [116]. Phylogenetic analyses of single nucleotide polymorphisms (SNPs) show significant intraspecific genetic variation in global metagenome samples [117]. There is considerable variation in the genomes of microbial strains, with the same species possibly using similar sequences for different functions [118, 119]. Therefore, to understand the role of genomic variation, editing with CRISPR‐Cas9 is a promising method to modify microbial isolates.

## SINGLE‐CELL GENOMICS AND TRANSCRIPTOMICS

Genome and transcriptome methods can achieve higher coverage and lower the risk of artifacts, but their assembly depends on the sequencing depth and community complexity [120]. For example, metagenomic approaches allow researchers to study bacteria in complex communities, but the complexity of these communities means that sequences of low‐abundance bacteria may be suppressed by those of higher abundance organisms, or, in the case of the human microbiome, by mitochondrial DNA [121]. Additionally, genetic information in cells with similar phenotypes may be significantly different and a large amount of low abundance information may be lost in the overall characterization. To make up for these limitations, single cell sequencing technology aims to reveal gene structures and gene expression of a single cell, better reflecting the heterogeneity between cells [122].

Looking at current publications analyzing the tumor microbiota using single cell techniques, there are four main research methods to study the genome and transcriptome: agar plate dilution [123], single‐cell exome sequencing based on fine capillary pipetting separation system coupled with an inverted microscope [124, 125], droplet‐based microfluidics [126], and improved fluorescence in situ hybridization (FISH) [127]. Notably, the first and key step of analyzing single cells is to prepare a single cell suspension from tissue or other sources.

### Agar plate dilution

**Figure 3:**
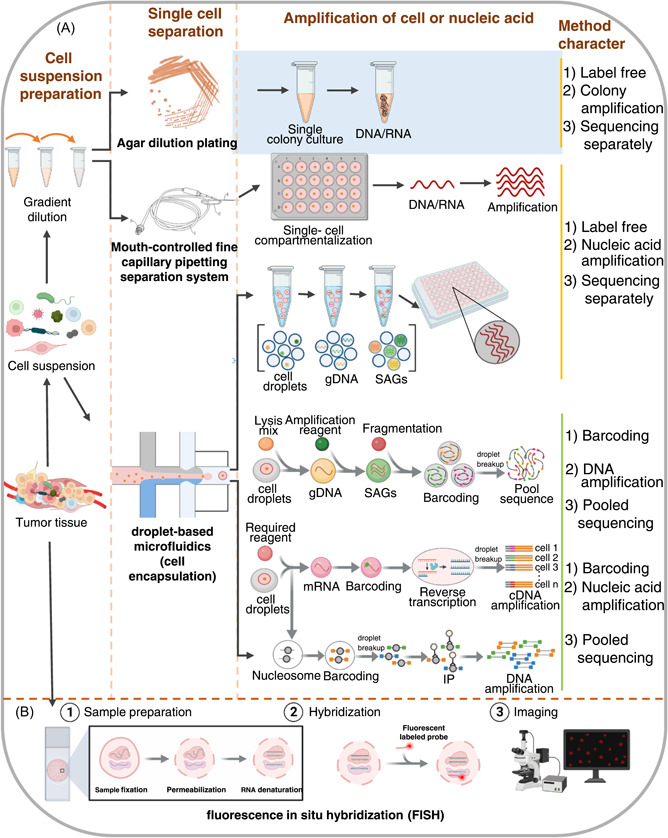
Single‐cell genomic and transcriptomic approaches. (A) Preparation of mixed cell suspension from tumor tissue (left). Workflow of three methods to separate mixture into single cells for genomic and transcriptomic sequencing (middle): agar dilution plating, mouth‐controlled fine capillary pipetting separation system, droplet‐based microfluidics (cell encapsulation). gDNA, guide DNA; SAGs, single‐cell amplified genomes. Comparison of characteristics of each method (right). (B) Workflow of single‐cell fluorescence in situ hybridization (FISH).

Agar plate dilution refers to the method of obtaining pure culture of a single microorganism by streaking plates and is the most traditional method of obtaining pure cultures of microorganisms. By diluting a mixture of microorganisms or different cell types repeatedly, one can separate diverse colonies into single cells, which will grow into homogenous colonies (Figure 3A). The development of the microfluidic streak plate (MSP) technique builds on traditional plate streaking with the use of microfluidic technology [128]. MSP uses microfluidics to make microdroplets that can be streaked onto dishes to grow single cells. Both traditional agar plate dilution and MSP can be integrated into the high‐throughput screening workflow.

### Methodology based on inverted microscope and mouth‐controlled fine capillary pipetting system

Serially diluted cell suspensions from tissues can be processed and isolated into single cells using a mouth‐controlled fine capillary system or other single‐cell pipetting systems under an inverted microscope. Isolated single cells are randomly isolated into PCR tubes and recorded as photomicrographs after visual confirmation with a microscope. Single‐cell genomes can then be extracted for whole genome amplification (WGA) [125]. This amplified genome can be used for exome sequencing, as described previously (Figure 3A). This method has been used to study the intra‐tumoral genetic landscape of renal cell carcinoma and adjacent renal tissues, finding that this carcinoma may be more complex than previously thought and promoting the use of more effective cell‐targeted therapies [124]. Notably, this approach may also be applied to study the microbes within individual tumor cells.

### Droplet microfluidic approach

The droplet microfluidic approach is mainly used to prepare droplets by using two incompatible liquids, with the generation of droplets being controlled by a microtubule structure and the flow rate ratio between the two liquids [120] (Figure 3A). Driven by a pump with a fixed flow rate, the two liquids enter different microchannels and when they meet, one fluid shears the other, creating micro‐droplets. These droplets are ideal micro‐reaction chambers and can be sized to accommodate a single cell. Individual droplets can be filled, manipulated, split, combined, detected, and classified with these systems, and thousands of individual droplets can be manipulated using microliters of reagents. Droplet microfluidics can separate a large population into individual cells, extracting and segmenting the genome through a series of enzymatic steps. Genetic material is amplified and marked before these single cells are sequenced. This is an integrated technology combining microfluidic technology, DNA barcoding, and sequencing technology.

An encapsulated microorganism can be directly cultured in liquid droplets and allowed to grow to hundreds of cells during the first genome amplification, before being lysed, and the genomic DNA purified. Then, a cell sorter is used to sort beads containing single‐cell amplified DNA into standard PCR plates, followed by reamplification into single‐cell amplified genome (SAG) library. A microfluidic droplet generator has been used to culture mouse gut microbes in agarose gel beads, a typical single‐cell sequencing method based on the SAG‐gel platform [120] (Figure 3A).

The encapsulated single microorganism can also be lysed directly, followed by DNA amplification or RNA reverse transcription and barcoding. For single cell genome sequencing, cells are usually amplified first, then labeled with barcodes [129, 130]. For single cell transcriptome sequencing, cellular RNA is usually labeled first, followed by reverse transcription, and finally complementary DNA (cDNA) amplification [131] (Figure 3A). To study nucleosomes within cells, the nucleosome must first be labeled with a barcode, followed by immunoprecipitation (single‐cell ChIP‐seq) and DNA amplification [132] (Figure 3A). Droplet microfluidic technology can analyze millions of independent reactions. Recently, it has been used for deep sequencing of a single DNA molecule, with the nucleosome being labeled to be analyzed through single‐cell ChIP‐seq and high‐throughput analysis [123, 133, 134].

The ability to sequence cells without having to separately culture microorganisms is a powerful aspect of micro‐droplet microfluidic single cell sequencing. With this method, a metagenome database can be generated to study microbes in tumors and other regions like the intestine. The limitation of this method are that it starts with a single genome copy and the loss of material during enzyme and microfluidics treatment is irretrievable, limiting the maximum available coverage and resulting in significantly lower coverage per cell.

### Improved fluorescence in situ hybridization (FISH)

FISH has always been an important tool to study cultured microorganisms, and can be directly applied to identify single bacterial cells, though traditional FISH has been limited by its low resolution. FISH includes the use of single‐stranded oligonucleotide probes to specifically label target RNA (Figure 3B). However, because the degree of fluorescence is directly related to the copy number of target RNA, this technique is most suitable for targeting rRNA. As different regions of 16 S rRNA have different levels of conservation, the probe can be species‐specific or selected according to different classification levels. To overcome low resolution, researchers have developed various methods, including catalyzed reporter deposition‐FISH (CARD‐FISH), also known as tyramide signal amplification (TSA) FISH, which uses horseradish peroxidase (HRP)‐labeled probes to amplify signals exponentially, so that single cells can be fluorescently labeled accurately and specifically [135, 136]. Highly phylogenetic resolution FISH (HiPR‐FISH) is another newly designed method that modifies existing algorithms in single cell automatic image segmentation and performs pixel classification and image optimization filtering [127]. Therefore, these advances have enabled researchers to better study specific microbial populations, visualize and sort small amounts of bacteria and very low copy RNA, and achieve single cell quantification [137]. It is likely that FISH will be used first to identify and locate microorganisms in tumor tissues, before they are sorted and studied using other analysis methods.

## PROTEOMICS

**Figure 4:**
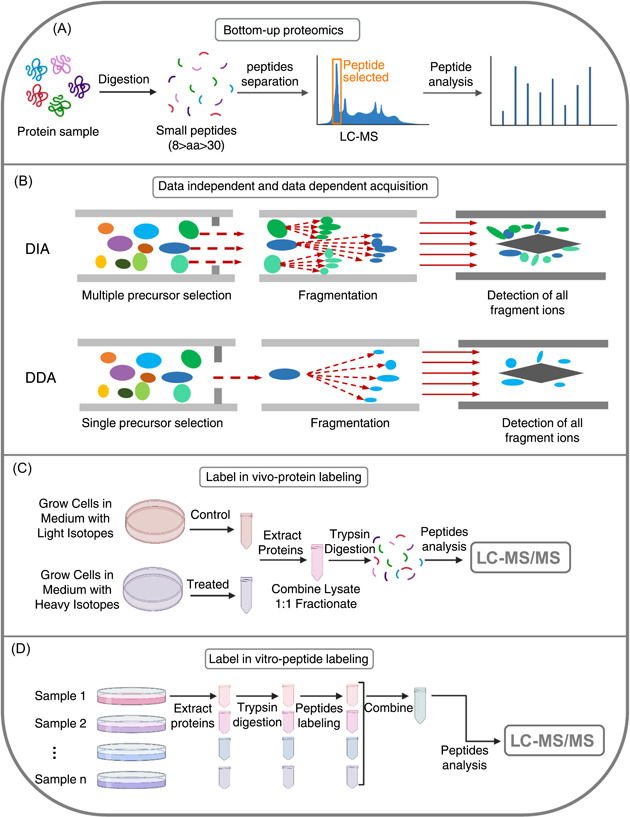
Different techniques of proteomics. (A) Procedure of bottom‐up proteomics. LC‐MS, liquid chromatography‐mass spectroscopy. (B) Data‐independent (DIA) and data‐dependent acquisition (DDA) modes of proteomics. (C) Workflow with SILAC (stable isotope labeling by/with amino acids in cell culture), a method of labeled quantitative proteomics, in vivo. (D) Workflow with TMT (tandem mass tag), a labeling quantitative proteomics method, in vitro.

Expression and regulation of the genome and transcriptome are directly reflective of tens of thousands of proteins, whose characteristics, including expression levels, PTMs, and protein‐protein interactions, are intricate. Mass spectrometry coupled with liquid chromatography (LC‐MS) is one of the most popular methods used for such analyses [138] and has become a powerful tool for tumor microbiome research [139–141]. There are three different strategies of proteomic analysis: “top‐down,” “middle‐down,” and “bottom‐up.” Specifically, “top‐down” (Figure 4A) [142] directly sends target proteins to mass spectrometry (MS) for analysis, without any pretreatment. “Middle‐down” [143] refers to proteins being partially digested to obtain relatively large peptide fragments, before being sent to MS for further analysis. Finally, “bottom‐up” analysis, also known as shotgun analysis, uses robust methods where the proteins are digested down to 6‐20 amino acids, before being analyzed by MS (Figure 4B) [144]. Among these three strategies, the third is the most widely used by proteome researchers globally, as the bottom‐up analysis strategy is usually more efficient for protein mixtures with high complexity that can contain thousands of mixed proteins of different orders of magnitude [145].

In proteomics, one of the major aims is to compare samples of interest (such as healthy and diseased tissue) to identify proteins that are differentially expressed and to quantify these differences. There are currently two broad approaches toward generating shotgun MS proteomics data: data‐dependent acquisition (DDA) and data‐independent acquisition (DIA) [146] (Figure 4B). In tandem with MS (MS/MS), the DDA strategy only selects certain peptides of higher signals generated in the first cycle of MS for fragmentation in the second cycle, while with the DIA approach, all peptides generated during the first MS cycle can be fragmented in the second round [147].

DIA has become the most popular choice in research on pathogenic mechanisms and biomarker screening with clinical samples, such as those of gut microbes [148] and tumors [149, 150], as it can obtain an unbiased and deeper look at the proteome of samples, particularly when these samples are from understudied organisms (e.g., tumor microbes) or complex tissues (e.g., tumor tissues). Compared to the DIA approach, DDA is a more widely applied and quantitative method in targeted analyses. Targeted proteomics analysis focuses on a subset of proteins of interest in a sample, mainly through parallel reaction monitoring (PRM) [151] or multiple reaction monitoring (MRM) [152]. MRM, also known as selective reaction monitoring (SRM), is a highly specific and sensitive MS technique that can selectively quantify peptides within complex mixtures. This technique uses a triple quadrupole MS (TQMS, QqQ) that first targets the peptide ion corresponding to the protein of interest, subsequently fragmentating it to produce a range of daughter ions. One (or several) of these fragmented daughter ions can be selected for quantification purposes. PRM is comparable to SRM/MRM, but is more convenient for assay development, due to its absolute quantification of proteins and peptides, based on high‐resolution and high‐precision mass spectrometry [153]. Protein quantification methods generally include absolute quantification and relative quantification. Absolute quantification determines the expression levels of the target peptide in the sample, following the establishment of a standard curve, for which the precise amount of the standard peptide is known [154]. Relative quantification determines fold changes in the expression between two samples by comparing intensities, without acquiring a standard curve.

There are labeled and label‐free methods of the relative quantification approach. Label‐free quantification methods aim to determine the relative amount of protein in two or more biological samples without using a stable isotope label bound to the protein. Commonly used labeled quantification techniques include isobaric tags for relative and absolute quantitation (iTRAQ) [155], tandem mass tag (TMT) [156], and stable isotope labeling by/with amino acids in cell culture (SILAC) [157], and are accomplished by labeling or substituting the corresponding amino acids with isotopic labels. TMT (Figure 4D) and iTRAQ are used for in vitro labeling, while SILAC is an in vivo labeling technology (Figure 4C). The advantage of these labeling methods is that the amount of proteins from different sources can be determined in a single experiment. The SILAC strategy stands out for substituting an isotopically heavy form of an amino acid for the naturally occurring light form. Because labeled and unlabeled samples are combined during the initial steps of sample preparation, SILAC minimizes quantitative error that may result from handling samples in parallel [158]. In addition, the mixing of samples permits a variety of enrichment techniques, including immunoprecipitation. These techniques can improve the detection of changes in abundance for both low‐abundance proteins and PTMs, such as phosphorylation and glycosylation [159–161].

## METABOLOMICS

**Figure 5:**
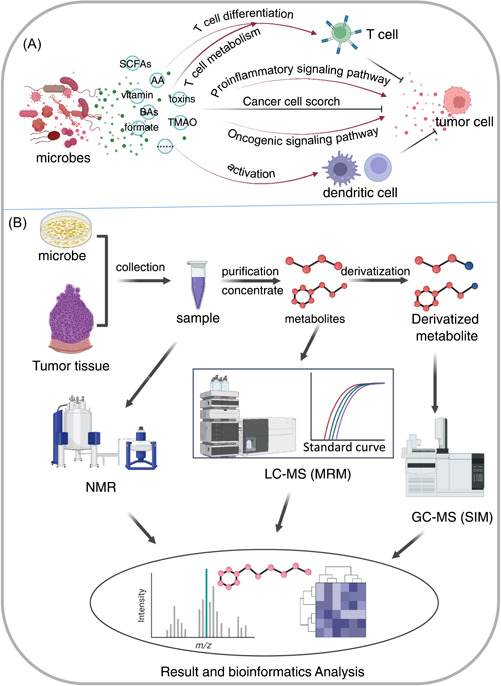
Metabolic methods used in tumor microbiome research. (A) Microbial metabolites affecting tumors through a variety of mechanisms. (B) Three most commonly used approaches of tumor microbe metabolomic research: nuclear magnetic resonance (NMR), liquid chromatography mass spectroscopy (LC‐MS) with multiple reaction monitoring (MRM), and gas chromatography mass spectroscopy (GC‐MS) with selection ion monitoring (SIM).

Metabolomics is widely applied to quantitatively analyze all the metabolites in different organisms and to discover the corresponding pathophysiological functions of these metabolites. The application of metabolomics in tumor microbiome research is committed to identifying the metabolic differences of tumor microorganisms and their interactions with the surrounding environment, to enhance the understanding of how tumor microorganisms affect molecular mechanisms related to carcinogenesis. These metabolites mainly include short‐chain fatty acids (SCFAs) [162], amino acid metabolites [163], vitamins [164], bile acids (BAs) [165], lactic acid [166], toxins [167], and formate [168], among others. Many studies have shown that microbial metabolites can significantly affect the occurrence and development of tumors through positive or negative regulation (Figure 5A), by affecting immunity, inflammation, signaling pathways, or by regulating the TME [26].

For example, SCFAs are one of the main metabolites of the intestinal flora. Studies have shown that SCFAs can affect intestinal function and intestinal metabolism positively and negatively. Supplementation of certain SCFA‐producing bacteria has been found to inhibit the development of intestinal tumors [169]. On the other hand, SCFAs are also expected to become a diagnostic and treatment target of CRC [169]. Isovaleric acid (IVA), a SCFA metabolite of intestinal microbes associated with CRC, can promote the expression of proteins that lead to downstream increases in 5‐hydroxytryptamine (5‐HT) [170]. 5‐HT directly acts on cancer stem cells, promoting self‐renewal and increasing intestinal tumor occurrence [171].

The interruption of bacteria producing vitamins B6 and B9 reduces the curative effect of 5‐fluorouracil (5‐FU), one of the most common chemotherapeutic drugs [172]. Additionally, Vitamin E secreted by intestinal bacteria can enter dendritic cells (DCs), enhancing the activation of DCs and promoting T‐cell antitumor immune responses [173].

Other metabolites of microbes can also regulate T‐cell metabolism, such as the secondary BA isoallolithocholic acid, which can enhance oxidative phosphorylation and mitochondrial ROS production, promoting T_reg_ differentiation [174]. Interactions between some refluxed BAs and gut microbes can lead to the production of the secondary BA taurodeoxycholic acid, which promotes the occurrence of gastric cancer [175]. Toxin‐producing bacteria *Clostridium difficile* can activate signaling pathways in intestinal epithelial progenitor cells, leading to increased production of ROS and tumor‐promoting mucosal immune responses, including the infiltration of activated myeloid cells and an increase in interleukin‐17 (IL‐17) producing lymphocyte subsets [176]. Formate secreted by *Fusobacterium nucleatum* can activate many signaling pathways, increasing invasiveness of CRC cells [168]. Trimethylamine oxide (TMAO) from intestinal flora enhances CD8^+^ T‐cell‐mediated antitumor immunity [177]. Gallic acid, another metabolite of intestinal microbes, has been shown to regulate T_reg_ cells and enhance cancer immunotherapy [176], while also causing a malignant phenotype in cells in the presence of p53 mutations [178]. Intestinal bacteria and their metabolites can promote tumors by inducing mutations, enhancing tumor‐promoting inflammatory responses, and recruiting immunosuppressive cells. Intestinal fungi can also induce a tumor‐promoting inflammatory microenvironment and can produce mycotoxins to induce mutations, thus promoting tumors [179].

### Analytical and sample preparation methods for tumor microbiome metabolomics

Metabolomics depends on the use of analytical techniques. Two instrument platforms (Figure 5B), nuclear magnetic resonance (NMR) [180] and MS [181], are the first choice at present. NMR is nondestructive, independent of analytical separation techniques, and the sample preparation is relatively fast, easy to operate, and low in cost. Absolute quantification using NMR can also be improved by adding an internal standard compound with a known concentration into the sample [182]. The principle of molecular characterization through NMR is in its strong magnetic field, which interacts with the nucleus of molecules, as they absorb and resonate the electromagnetic spectra of a specific frequency. However, due to the relatively low sensitivity of NMR, complexity of microbial metabolites, and variation in metabolite concentrations, the application of NMR in microbial metabolomics is limited [183].

To overcome these problems, high‐resolution MS has become an indispensable tool in metabolomics research. The technology utilizes various separation techniques, including liquid chromatography (LC), gas chromatography (GC), and capillary electrophoresis (CE), and is widely used in microbial metabolomics [184]. GC‐MS is one of the most commonly used technologies in metabolomics, due to its stable performance, good reproducibility of results, and high peak capacity [185]. This technique is mainly used for volatile and thermally‐stable low molecular weight compounds. However, most microbial metabolites, such as phosphorylated metabolites, are non‐volatile and thermally unstable, and thus, may be degraded when placed at higher temperatures. Therefore, this method is often used to analyze derivatized microbial metabolites, such as those from *Propionibacterium fischeri*, *E. coli*, and *Bacillus subtilis* [186]. Miyamoto et al. has also utilized GC‐TOF‐MS to find biomarkers associated with lung cancer prognosis [187]. CE‐MS is another tool for metabolomics analysis [188]. Compared to GC‐MS and LC‐MS, its advantages include high separation efficiency, small sample volume, and low cost. However, its main disadvantage is that the interface between CE and MS is difficult, so CE‐MS is not widely used. LC‐MS and tandem LC‐MS/MS are important analytical tools in metabolomics. Unlike GC‐MS, LC‐MS can analyze polar and high molecular weight compounds without high temperature or volatility, has a wider coverage of metabolites for analysis, and has more convenient sample preparation [189]. In addition, LC‐MS has a higher sensitivity, even with a small number of samples [190]. The appearance of ultrahigh performance liquid chromatography (UHPLC) has greatly improved the chromatographic resolution of LC‐MS.

Metabolomics research generally falls into two categories: targeted metabolomics and nontargeted metabolomics [191]. Nontargeted refers to the systematic analysis of all metabolites in a sample, while targeted refers to the relative or absolute quantification of metabolic changes, with absolute quantification often using SRM and an established standard curve. TQMS and traditional ion trap mass spectrometers have been widely used in targeted metabolomics research. However, the implementation of nontargeted metabolomics has required more advanced analytical methods, automated spectral data processing, biological data interpretation, and hypothesis generation [192]. High‐resolution tandem mass spectrometers, such as quadrupole time of flight (Q‐TOF) mass spectrometers, Fourier‐transform ion cyclotron resonance (FT‐ICR) mass spectrometers, and ion trap mass analyzer Orbitrap‐based mass spectrometers, are the preferred instruments for nontargeted metabolomics research, as they can clarify structural information and allow for high data acquisition speed [193].

Intracellular metabolism can change rapidly based on time and the external environment [194]. Therefore, methods and conditions of sampling and sample preparation, including time, storage conditions, and other factors, can greatly affect the reproducibility, precision, and accuracy of metabolic detection. Sample preparation occurs in two stages: metabolic quenching and extraction of metabolites. Quenching is the process of rapidly stopping the metabolic activity of cells with limited cell membrane damage [195]. Tissues or cells are collected quickly, immediately frozen in liquid nitrogen, and stored them at −80°C. In addition, other methods like freezing quenching or chemical quenching with acids or cold aqueous methanol solutions are also commonly used.

Extraction is the process of separating metabolites from samples. Extraction can be achieved through physical means, by manual grinding or using a tissue homogenizer. Solvent extraction using organic solvents, such as methanol, chloroform, and acetonitrile, is the more commonly used method, because of its higher extraction efficiency and minimal ionization suppression in LC‐MS analysis [196]. NMR‐based analysis does not need chemical derivatization and the sample preparation is much simpler. Both of these microbiologic metabolic analysis methods have advantages and disadvantages. Researchers should choose the best method or combination of tools to analyze microbial metabolites, according to the characteristics of interested compounds and availability of analysis tools.

## SINGLE‐CELL PROTEOMICS AND METABOLOMICS

Single‐cell sequencing is used for the analysis of the cellular genome, transcriptome, and epigenome. Similarly, single‐cell proteome and metabolome studies are also essential for genome and transcriptome research on single cell levels. Unlike single‐cell nucleic acid sequencing where signals can be amplified, single‐cell protein and metabolite analysis signals cannot be. In this type of research, tracing is the biggest challenge, as this is critical to reduce the loss of intracellular analytes during sample processing.

The field of single‐cell proteomics is still in its infancy, and lacks a clear and generally applicable approach [197]. Nonetheless, recent rapid progress in absolute quantification of single‐cell proteins and highly multiplexed protein measurements has relied on the increased sensitivity of MS instruments and the improvements in sample preparation methods to overcome the limitations of traced proteins in single cells.

Orbitrap‐based high‐resolution MS instruments have undergone continuous improvements in ion transmission efficiency, detector sensitivity, duty cycle, and resolution over successive generations of mass spectrometers. Recently, when individual HeLa cells were analyzed on the latest generation of the Orbitrap Eclipse MS, peptide and protein coverage increased by 36% and 20%, respectively [198]. The latest MS instruments have also introduced drift tube‐based ion mobility spectrometry (DT‐IMS) [199, 200], trapped ion mobility spectrometry (TIMS) [201], and high field asymmetric waveform ion mobility spectrometry (FAIMS) [202], which can improve selectivity, separate or filter singly charged ions from multiply charged peptides, and select multiply charged peptides for self‐determination. In terms of identification and quantification with shotgun proteomics, the removal of singly charged species is beneficial for trace sample analysis, since solvent clusters and contaminants that are negligible in batch studies can become very important in low‐input studies. In all cases, LC separation and MS acquisition settings, such as column, flow rate, ion injection time, and automatic gain control (AGC) target settings, in single‐cell analyses tend to be very different from those used for batch studies, ensuring that sufficient reporter ion signal is available for accurate quantification and maximum ion utilization. However, current techniques of single‐cell metabolomics have mainly utilized single‐cell analyses of cultured microbes, and cannot be directly applied to multicellular organisms to study microbe‐cell interactions [203]. Mass spectrometry imaging (MSI) has shown great potential in the field of single‐cell metabolism, by mapping metabolites and other biomolecules in tissues [204].

Matrix‐assisted laser desorption/ionization (MALDI) mass spectrometry, a type of MSI technology, is currently another one of the most commonly used single‐cell metabolic analysis techniques [203, 205–207]. A sample is mixed with a matrix and irradiated with a pulsed laser. The beam protonates or deprotonates the analyte, before sending it into the mass spectrometer. MALDI‐MS measures mass spectra at different points in tissue sections or slides containing plated cells, allowing researchers to create spatial maps of metabolism. MS images using MALDI have been obtained with a spatial resolution below 5 μm [208, 209], which has made studying the molecular distribution of metabolites more viable. MALDI‐MS stands out for requiring less sample preparation and having a higher throughput than other methods, and has been successfully used to reveal the heterogeneity among clonal populations of single‐celled organisms and discover specialized microorganisms and cellular subtypes in tissues [210–212]. The two biggest limitations of MALDI imaging are low metabolite coverage and low ionization efficiency. To overcome low metabolite coverage, Feenstra et al. proposed an MSI approach of using multiple matrixes with different ionizations to target dual polarities, alongside tandem MSI, though the metabolites identified with low ionization energies were still limited [213]. The ionization efficiency can be increased by on‐tissue chemical derivatization [214, 215]. The newer and more sensitive MALDI‐2 was developed to have an enhanced analyte signal through the initiation of an additional ionization process in the gas‐phase laser plume produced via a second laser [216].

These advances suggest that improvements to MSI can increase our understanding of different metabolites at the cellular and subcellular levels. As an evolving technology, MSI still has many challenges to overcome. The data obtained from MSI is complex and can contain signals from known and unknown metabolites [217]. Improvements in software and databases are essential to identify metabolites with a high degree of confidence and to draw accurate biological conclusions. Furthermore, advances in quantification methods will be critical to distinguish results that may be biased by matrix effects or analyte ionization efficiency.

## APPLYING MULTI‐OMICS APPROACHES FOR TUMOR MICROBIOME RESEARCH

We are growing more aware that a single field of omics is not comprehensive enough to understand more complicated biological processes, such as those related to the tumor microbiome. Integrated multi‐omics seeks to fill this gap in research and is a potential new direction to explore these topics.

It has been shown that a variety of cytotoxic metabolites produced by microbes can play a vital role in tumorigenesis and tumor development. Using the metabolite methylglyoxal (MGO) as a case study, MGO is known to affect human health in various manners. MGO, a nonenzymatic byproduct of aerobic and anaerobic glycolysis [218, 219] and a microbial metabolite enzymatically synthesized by MGSs [25], can covalently modify proteins without enzymes and regulate cell functions, including metabolism and transcription [79, 220–223]. It has been reported that MGO can modify DNA and RNA by reacting with guanine residues, inducing DNA and RNA damage [218, 220], and directly affect the integrity of the genome by damaging chromosome segregation during mitosis [224]. Additionally, MGO can react with lysine residues of proteins, leading to the formation of advanced glycation end products (AGEs), which in turn, can cause cellular dysfunction and inflammation [224]. MGO is one of the most effective glycating agents in vivo and can glycate the lysine and arginine residues of histones, which can lead to metabolic disturbances, epigenetic changes, and instability of nucleosomes [219–221, 224, 225]. It has been reported that MGO can affect the activity of transcription factors and change gene transcription, as seen through RNA sequencing, and can lead to the accumulation of cell stress factors [221]. The two‐stage model of histone MGO glycosylation suggests that a low amount of MGO is beneficial to the proliferation of cancer cells by promoting complex transcription, while an excessive amount of MGO leads to chromatin cross‐linking, weakening transcription and leading to cell death [221].

**Figure 6:**
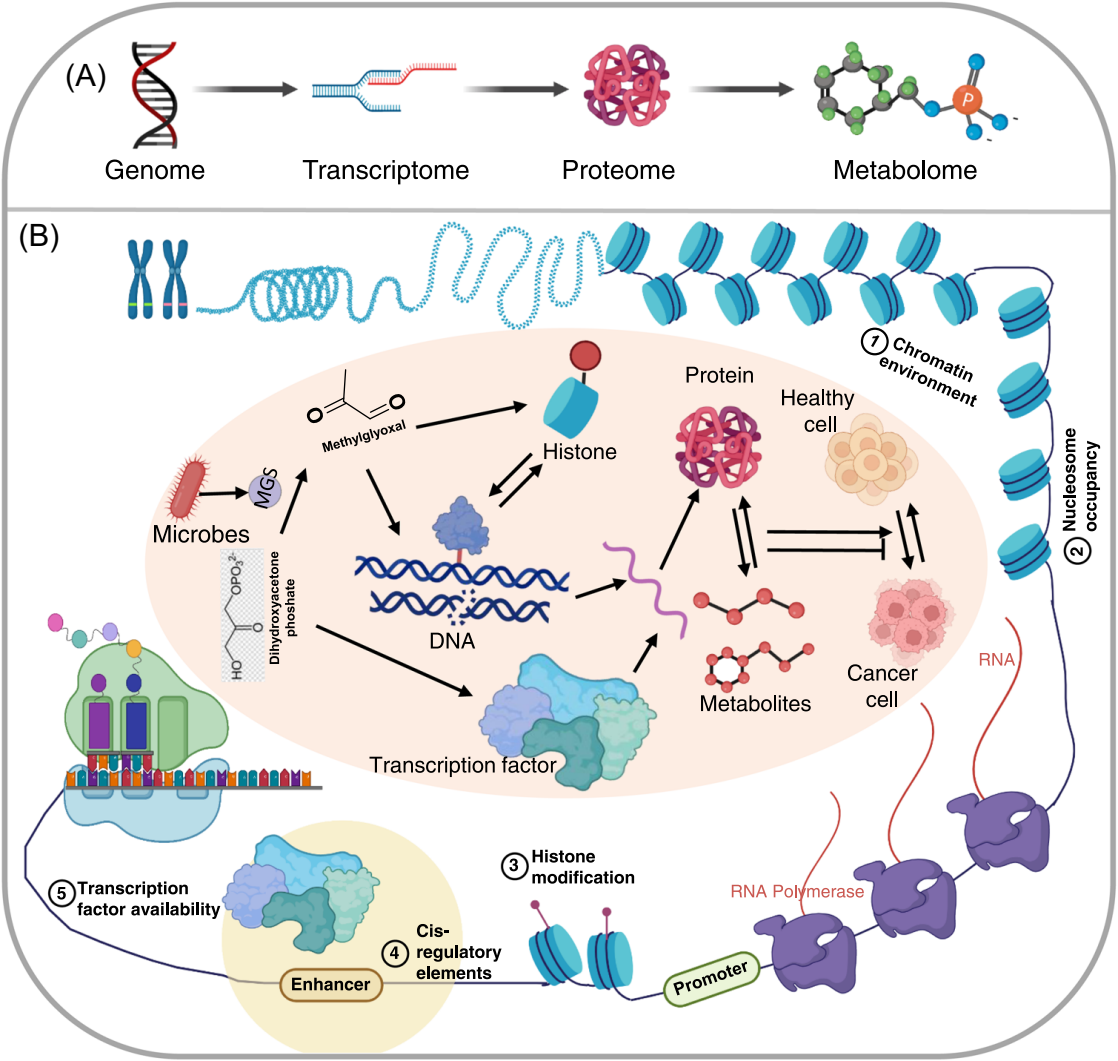
Multi‐omics allows for a better understanding of interrelated disciplines. (A) Flow of omics techniques, which can be combined for multi‐omics, through layers of the central dogma. (B) The metabolite methylglyoxal can affect many levels of the multi‐omics network, including genes, proteins, histones, and chromatin. All of these pathways can contribute to tumor development. MGS, methylglyoxal synthase.

Chemical proteomics methods have shown that high concentrations of MGO can induce histone‐histone and histone‐DNA cross‐linking in chromatin, which can impair its kinetic properties [220]. The synthesis and application of chemical probes for the study of MGO‐based glycation can improve the accessibility and feasibility of important tools for tracking, enriching and studying MGO‐based glycation, and advance the understanding of its potential biochemical functions [226]. MGO regulates the occurrence and development of tumors at genomic, transcriptomic, proteomic, and metabolomic levels (Figure 6). Research on MGO has shown that using a single omics method is not enough to comprehensively understand the effects of such proteins in biological research, and presents a case study for other metabolites and proteins that may require a more interdisciplinary multi‐omics approach.

## FUTURE PERSPECTIVES

Tumor microbes have been shown to play an essential role in carcinogenesis, development, and treatment [227–229]. The interactions between microbes, tumor cells, and immune cells can greatly affect the TME. Immune cells not only influence the relationship of microbes to anticancer therapies [230], but can also directly modulate the influence of microbes on cancer development [231]. Based on this, combining microbial therapy with immunotherapy has become an effective direction for cancer treatment research [232]. Multi‐omics techniques have been applied to identify cancer biomarkers produced by the tumor cells and microbes in the TME [39, 233, 234], thereby significantly facilitating the development of new diagnostic and treatment strategies. Although many advances have been made in the field through omics approaches, the field is still in its infancy. Many research techniques are immature, such as the application of single‐cell technologies in tumor microbial proteomics and metabolomics research, which is still largely theoretical. Moreover, there is a lack of a unified standard process for multi‐omics studies of the tumor microbiome. All in all, in this review, we have systematically summarized the muti‐omics approaches that can be applied for tumor microbiome research, as well as their advantages and shortcomings. Due to the low biomass of intratumoral microbes and the heterogeneity of TME, we propose that single cell muti‐omics will become the most powerful tool in tumor microbiome research, though there is still a long way to go to develop the corresponding methodologies.

## AUTHOR CONTRIBUTIONS

Nan Zhang drafted the manuscript, made the figures, and edited the references. Shruthi Kandalai edited the manuscript and helped to edit the references. Xiaozhuang Zhou helped to make Figure 4 and assisted to edit the references. Farzana Hossain helped to edit the references. Qingfei Zheng proposed the conception, wrote, and edited the manuscript. All authors listed in the paper have made a substantial, direct, and intellectual contribution to the work and approved it for publication.

## CONFLICT OF INTEREST

The authors declare no conflict of interest.

## Data Availability

No new data or script was used in this paper. Supplementary materials (figures, tables, scripts, graphical abstracts, slides, videos, Chinese translated versions and updated materials) may be found in the online DOI or iMeta Science http://www.imeta.science/.
